# Lectin Protein as a Promising Component to Functionalize Micelles, Liposomes and Lipid NPs against Coronavirus

**DOI:** 10.3390/biomedicines8120580

**Published:** 2020-12-07

**Authors:** Mehran Alavi, Kofi Asare-Addo, Ali Nokhodchi

**Affiliations:** 1 Nanobiotechnology Laboratory, Faculty of Science, Razi University, Kermanshah 67146, Iran; 2 Department of Pharmacy, University of Huddersfield, Queensgate, Huddersfield HD1 3DH, UK; k.asare-addo@hud.ac.uk; 3 Pharmaceuics Research Laboratory, Arundel Building, School of Life Sciences, University of Sussex, Brighton BN1 9QJ, UK

**Keywords:** coronaviruses, COVID-19 pneumonia, lectins, nanoformulations, micelles, liposomes

## Abstract

The outbreak of a novel strain coronavirus as the causative agent of COVID-19 pneumonia, first identified in Wuhan, China in December 2019, has resulted in considerable focus on virulence abilities of coronavirus. Lectins are natural proteins with the ability to bind specific carbohydrates related to various microorganisms, including viruses, bacteria, fungi and parasites. Lectins have the ability to agglutinate and neutralize these pathogeneses. The delivery of the encapsulated antiviral agents or vaccines across the cell membrane can be possible by functionalized micellar and liposomal formulations. In this mini-review, recent advances and challenges related to important lectins with inhibition activities against coronaviruses are presented to obtain a novel viewpoint of microformulations or nanoformulations by micellar and liposomal cell-binding carriers.

## 1. Introduction

**Figure 1:**
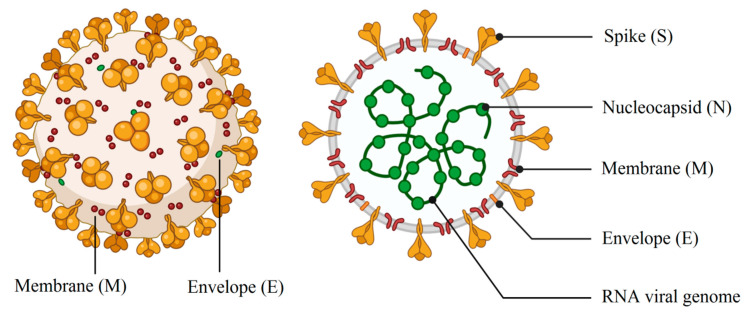
Schematic drawing of main components of spherical or pleiomorphic coronaviruses (credit: biorender.com).

Coronaviruses, a large family of RNA viruses (a positive sense, single-stranded RNA in the range size of 27–32 kb), belong to the subfamily Orthocoronavirinae in the family of Coronaviridae in the order Nidovirales [1]. The genome of the coronavirus is composed of open reading frames (ORFs), the first of which comprises two-thirds of the genome and encodes the replicase proteins. The last third contains the structural protein genes in a fixed order [2]. These viruses may cause diseases in birds and mammals, such as the infectious bronchitis virus (IBV) and feline coronavirus (FCoV), respectively [3,4,5]. The 1930s brought about the discovery of the first member of the family of the coronavirus [6]. The severe acute respiratory syndrome (SARS) outbreak in 2002–2003 shook the world and brought this to the forefront of research, and thus the discovery of more of the members of this family of viruses after the epidemic [2]. As illustrated in Figure 1, a complete viral particle is composed of several proteins including nucleocapsid (RNA + nucleoprotein), spike, envelope and the membrane proteins [7]. It is important to note that some viruses may contain hemagglutinin esterase (Figure 1). The membrane and envelope proteins are involved in virus assembly, whereas the spike protein is the leading mediator of viral entry and the principal player in determining host range [2]. The 3C-like protease and the papain-like protease (PLP) are the main viral proteases related to coronaviruses [5]. In humans, severe acute respiratory syndrome (SARS), Middle East respiratory syndrome (MERS), and coronavirus disease 2019 (COVID-19) can lead to health-threatening diseases. The novel virus, severe acute respiratory syndrome coronavirus 2 (SARS-CoV-2), has led to COVID-19. The outbreak of this novel strain of coronavirus as the causative agent of COVID-19 pneumonia, first identified in Wuhan, China in December 2019, has resulted in considerable focus being placed on coronavirus virulence properties [8]. The most severe form of this novel virus has been reported to lead to hypoxia and acute respiratory distress syndrome (ARDS), which can lead to the requirement of invasive mechanical ventilation [9]. Many countries in the Middle East, Europe and the United States have been affected by this infection. Based on recent reports, COVID-19 can be classified as a mild, moderate and severe disease. The fatality rate of 2% up to 2.5% and the acute disease are as a result of severe alveolar damage and respiratory failure [8,10].

A viral infection is dependent on the interaction of viral particles with specific receptors on the cell membrane. The fusion of the virus envelope with the cell membrane is mediated by the spike glycoproteins [2]. Furin is one of the proteases with high expression in lung cells which can be responsible for the proteolytic cleavage of the envelope proteins of viruses such as SARS-CoV and human immunodeficiency virus (HIV). In SARS-CoV-2, the furin-like cleavage site in the spike glycoprotein may contribute to the pathogenicity and the viral life cycle [11].

This review seeks to address the recent advances and challenges related to important lectins with inhibition activities against coronaviruses. This is carried out to aid the reader to obtain a novel viewpoint of microformulations or nanoformulations by micellar and liposomal cell-binding carriers.

## 2. Antiviral Therapies

Several anti-infective agents, such as chlorine-containing disinfectant, 62–71% ethanol (ethyl alcohol), 0.5% hydrogen peroxide or 0.1% sodium hypochlorite (bleach) and exposure to 56 °C for 20 min or 65 °C for 10 min in serum medium, may be used to deactivate or remove virus [12]. According to patients’ conditions, synthetic antiviral drugs, including α-interferon oral spray, interferon-β, lopinavir/ritonavir, favipiravir, remdesivir, ribavirin, corticosteroids, chloroquine phosphate, and arbidol, may be prescribed for acute cases of COVID-19 [13,14,15,16,17,18,19]. Current antiviral agents can target entry, fusion, viral RNA, and proteases of coronaviruses. α-interferon has been reported to inhibit SARS-CoV reproduction in vitro [20]. Lopinavir/ritonavir has been reported to have anti-SARS-CoV activity in vitro as well as in clinical studies [21]. Chloroquine has also been reported to have the ability to block SARS-CoV-2 infection at low micromolar concentrations [15]. Hydroxychloroquine has been used to prevent and treat COVID-19 via blocking the interactions between the virus and the angiotensin-converting enzyme-2 receptor, as well as the sialic acids receptor. Its use has been found to be effective in vitro [22,23] and in preliminary clinical results [24,25]. The main hurdle is that the concentration of hydroxychloroquine in the lung is not good enough to kill coronavirus when administered via the oral route. Therefore, an inhalation formulation which directly delivers the drug to the respiratory tract whilst minimizing the systemic exposure would be a desirable alternative [26]. Tai et al. showed that the liposomal formulation of hydroxychloroquine can be more effective via the inhalation route compared to the injection or oral route in tackling COVID-19. The authors showed that the liposomal formulation produced high concentrations of hydroxychloroquine in the lung (~30-fold) with a longer half-life (~2.5-fold) when the novel liposomal formulations were administered by intratracheal instillation in Sprague–Dawley (SD) rats as compared to the intravenous formulation [27]. It was concluded that the inhalable liposomal formulation containing hydroxychloroquine may provide clinical benefits and serve as a potential treatment for COVID-19. In another study carried out by Feliciello and Procino [28], the authors showed that the pulmonary proteoliposome as a new therapeutic approach could be effective in reducing the overall viral load in the lung, thereby helping the immune system to reduce the lung infection. There are, however, various dose-dependent side effects such as cardio-metabolic, hypercholesterolemia, hypertriglyceridemia, and adverse gastrointestinal (GI) effects for this type of antiviral therapy [29,30]. Some of the most common GI effects reported for lopinavir/ ritonavir includes diarrhoea and nausea [31]. The efficiency of vitamins A, E, D3, C and B in the prevention and reduction of coronaviruses-associated diseases is reported by several studies [32,33]. Vitamin A and retinoids have been reported to inhibit measles replication by upregulating elements of the innate immune response in uninfected bystander cells. During subsequent rounds of viral replication, this, therefore, makes them refractory to productive infection making it promising for the treatment of SARS-CoV and the prevention of lung infection [34]. It has been reported that vitamin B2 and UV light in combination was effective in reducing the titer of MERS-CoV in human plasma products [33]. Vitamin B3 has been reported to be efficacious in both prophylactic and therapeutic settings [35] and also inhibited neutrophil infiltration into the lungs, whilst demonstrating anti-inflammatory effects from lung injuries induced by ventilators [36]. In addition, the application of natural compounds extracted from herbal metabolites can reduce these side effects owing to biocompatibility and biodegradability of these materials. Herbal materials such as betulinic acid, lignin [37], coumaroyltyramine, quercetin, and N-cis-feruloyltyramine [38] have shown antiviral activities against MERS and SARS. 3C-like protease and papain-like protease were targeted for these herbal compounds [39]. There are several ways for inhibiting the coronavirus entry into target cells. Binding inhibitors to angiotensin-converting enzyme-2 (ACE2) receptors is the first way [40]. For instance, a recent study showed that human recombinant soluble ACE2 (hrsACE2) can hinder the early stages of SARS-CoV-2 infections significantly [41]. These receptors are single-pass type I membrane proteins with a higher expression on the surface of epithelial cells of the pulmonary alveolus, vascular endothelial cells of the heart and the kidneys, and small intestine enterocytes. The S1 domain of the spike protein of SARS-CoV can bind these metallopeptide receptors [42]. In this regard, patients with hypertension, cardiac diseases and diabetes may experience severe COVID-19 infection owing to the use of ACE2-enhancing drugs [43]. The second type of inhibitors bind to the coronavirus and block its interaction with related receptors on the cells. Prevention of conformational changes and fusion of viruses with the cells represents the third class of inhibitors [40].

## 3. Lectins

Antitumor, antiviral and antimicrobial activities of lectins have been reported by various studies [44,45,46]. Before the initiation of an antibody’s activity in the body, the mannose-binding lectin (MBL) as a humoral protein can block the entry of coronavirus into the target cells [42,47,48]. Moreover, MBL increases viral neutralization by the activation of the complement system and the influx of innate immune cells [49]. The innate immune system employs this mechanism to hinder viral and bacterial infections in alveolar cells. Surface-active phospholipoprotein complexes containing phospholipids (80%), cholesterol (10%) and proteins (10%) with the names of SP-D, SP-C, SP-B, and SP-A are secreted by type II alveolar cells. SP-A and SP-D collectins having C-type lectins can opsonize bacterial and viral pathogens and facilitate the phagocytosis action by monocytes and macrophages. Interaction of collectins with carbohydrate moieties of spike protein on the surface of the SARS-CoV can lead to inactivate viral uptake and reducing pulmonary inflammation, wherein a mild and moderate illness in smokers may be resulted from increased levels of SP-A protein levels in sputum, plasma and the serum [50].

**Figure 2:**
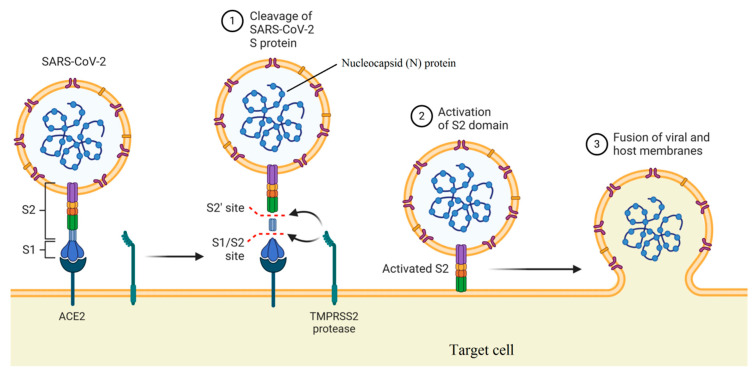
Mechanism of viral entry by function of two subunits of the spike protein (created in BioRender.com).

Natural carbohydrate-binding proteins such as lectins have demonstrated antiviral properties in the case of HIV and coronaviruses [51,52]. In this case, the spike glycoprotein related to SARS coronavirus and the envelope glycoprotein GP120 of HIV is the main targets for a red algae-derived lectin (griffithsin) [52,53]. In addition, griffithsin has antiviral activities against the hepatitis C virus [54]. Plants express twelve different families of lectins with binding ability to mono- or oligo-saccharides related to glycolipids or glycoproteins [55,56]. As illustrated in Figure 2, two functional subunits including S1 and S2 subunits are recognized for spike protein, which facilitates binding to the receptors of the host cell and fusion of viruses with the cellular membrane. The spike protein with a trimer structure is decorated with N-linked glycans that are critical components for appropriate folding and neutralizing by specific antibodies and host proteases [57]. The high-mannose oligosaccharides related to spike and membrane glycoproteins can be targeted by herbal lectins, wherein two coronaviruses, namely, feline infectious peritonitis virus and mouse hepatitis virus, were blocked by *Galanthus nivalis* agglutinin (GNA), *Urtica dioica* agglutinin (UDA), and Hippeastrum hybrid agglutinin (HHA) via the inhibition of virus entry at a post-binding stage [58].

According to structure type, there are four lectins, namely, super lectins (with dissimilar carbohydrate-binding domains), chimero lectins (hybrid proteins composed of one or more carbohydrate-binding sites), hololectins (at least two carbohydrate-binding domains) and merolectins (single carbohydrate-binding site). Almost all agglutinating lectins are located in the hololectins group [59]. A reduction in the intracellular loading of RNA associated with SARS-CoV was observed for the MBL of the amaryllis plant species after 8 h of infection [40]. Red, brown, and particularly green alga (nearly 500 types of lectin) are the main sources of algal lectins. Self-assembly of viruses may be disrupted by the effects of MBL [60]. More antiviral activities of MBL (extracted from red algae Grateloupia chiangii) toward the herpes simplex virus was observed compared to the influenza virus. In this case, MBL had a significant ability to bind maltoheptaose-β-Sp1 and maltohexaose-β-Sp1 [44].

## 4. Micelles and Liposomes

**Figure 3:**
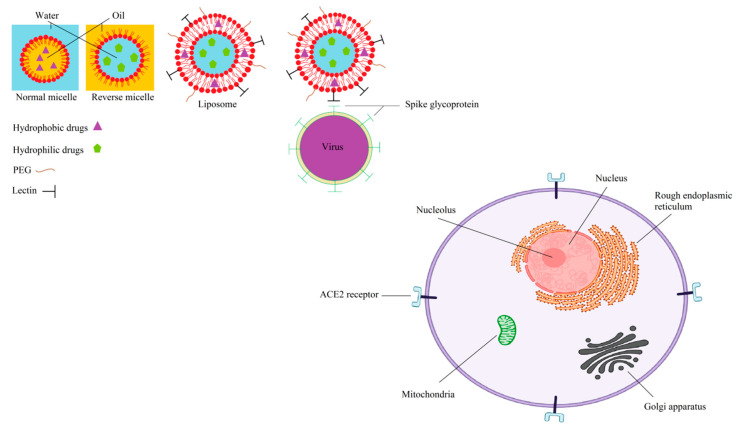
Schematic images of a normal micelle, reverse micelle, and liposome with common (PEGylation which is the modification of a protein, peptide or non-peptide molecule by the linking of one or more polyethylene glycol chains) and particular (by lectins) functionalization as well as the interaction of the lectin-modified liposome with spike glycoprotein of virus (only the cell image is extracted from the biorender.com website; the rest are drawn by the authors).

Nanotechnology has presented new insights in various medicinal fields particularly in the case of infectious diseases therapy [61,62,63,64]. Nanomaterials (NMs) are in the size range of 1–100 nm, and their unique physicochemical properties are a vital element of this technology. Large surface areas and higher reactivates are expected for NM compared to bulk one [65,66,67,68]. Nanoparticles (NPs), nanowires (NWs) and nanoplates (NPLs) are zero, one, and two-dimensional examples of NMs [69,70,71]. Self-aggregation of surfactants in the liquid colloidal medium under specific conditions of surfactant concentrations (more than critical micelle concentration) and temperature of the system (higher than Krafft temperature) results in micelles formation. As presented in Figure 3, lipid micelles have a single layer of lipid monolayers with fatty acid and aqueous cores for the respective oil in water (O/W) and water in oil (W/O) types [72,73]. Based on the physicochemical properties of an antiviral or an immune-stimulant agent, either the surfactant layer or the core part may be used to load them [74].

Liposomes in the nanoscale, as one type of NP, are prepared by the formation of a phospholipid bilayer vesicle in an aqueous medium (Figure 3). Single bilayer and multilayer liposomes are the two main types of liposome [75,76]. Both the bilayer and the aqueous core of this carrier may be used in the encapsulation of antiviral drugs [77,78]. In addition, loading via absorption or anchoring on the liposome surface can be applied for some therapeutic agents or antibodies [79]. In contrast to passive liposomes, active liposomes have been prepared by the targeted functionalization of the surface of liposomes for specific targets [80,81]. For example, a common modification of a liposome surface is through the use or application of polyethylene glycol (PEG) to increase water solubility and thus decrease the renal clearance of the liposome and immunological responses in physiological conditions (Figure 3) [82]. Delivery of the encapsulated antiviral agents across the cell membrane can be possible and achieved by liposomal formulations. In addition to drug entrapment efficiency, size variation and stability of the formulation, biocompatibility and biodegradability in physiological conditions, particularly in pulmonary tissues, are vital factors to treat COVID-19. For example, in the preparation of liposomes by the heating method compared to conventional methods, such as the ethanol injection, anionic liposomes displayed homogenous size ranges, stabilities for more than eight months, and no toxicity for the 400 nm and 100 nm diameter toward the human bronchial epithelial cell line [83].

**Figure 4:**
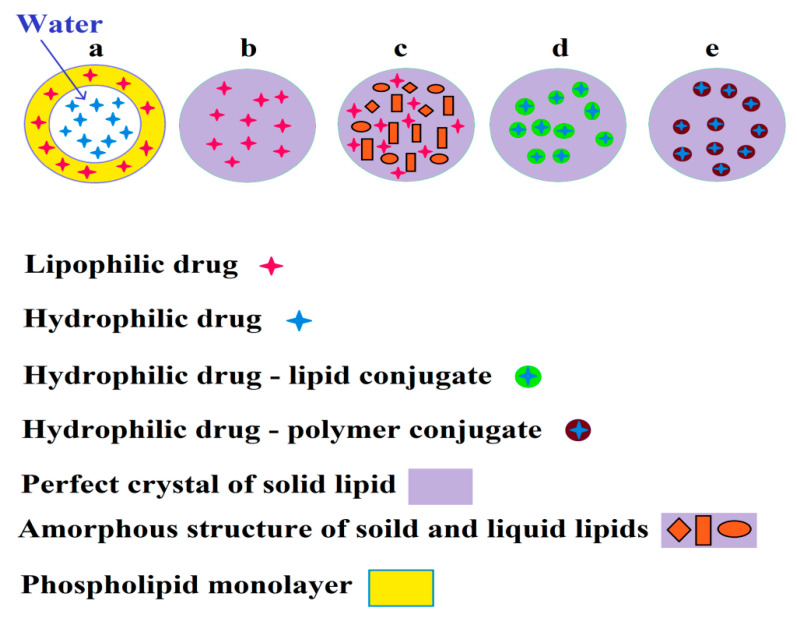
Encapsulation of lipophilic and hydrophilic drugs by a liposome (a), solid lipid nanoparticles (b), nanostructure lipid carriers (c), lipid–drug conjugates (d), and polymer–drug conjugates (e).

Solid lipid NPs (SLNs), nanostructure lipid carriers (NLCs), lipid–drug conjugates (LDCs), and polymer–drug conjugates (PDCs) are other lipid-based NPs, which can be employed to load and encapsulate antiviral agents (Figure 4) [84]. Specific advantages involving biodegradability, biocompatibility, low degradation of the drug in physiological conditions owing to encapsulation, efficient drug loading, increased half-life of drug, controlled release and sustained release of therapeutic agents, improved drug absorption and dissolution, desirable bioavailability, passive and active targeting capacity, simple sterilization, and large scale production are demonstrated by these colloidal carriers [85,86].

In the following section, some important micellar, liposomal, and lipid NP formulations are presented as novel carriers for encapsulation and loading of antiviral agents. This may be useful in obtaining a new viewpoint about microformulations and nanoformulations of antiviral agents against health-threatening diseases caused by coronaviruses particularly COVID-19 pneumonia.

## 5. Micellar, Liposomal, and Lipid NP Formulations

Mixed micelles are formed by the self-assembly of two different di- or tri-block copolymers of surfactants in a colloidal medium [87]. The bioavailability of poorly water-soluble drugs can be augmented by polymeric mixed micelles. Efavirenz (EFV) is a non-nucleoside reverse transcriptase inhibitor which is recommended as an oral administration with other antiviral agents against HIV. Recent information suggests that this drug may inhibit SARS-CoV-2 by a combination with other antiretroviral drugs [88]. The high hydrophobic property of this drug leads to a lower bioavailability and dose-dependent side effects on the central nervous system (CNS). Loading of EFV by mixed micelles prepared by the use of Pluronic^®^ F127 and Tetronic^®^ T904 showed the enhanced bioavailability of this drug by four folds [89]. Viral RNA replication and translation are inhibited by a camptothecin drug as a topoisomerase inhibitor [90]. A polymethacrylate block containing 2,6-diacylaminopyridine pendant (DAP) units and a PEG (at 2000 (PEG2) and 10,000 (PEG10) molar masses) were utilized to encapsulate this hydrophobic drug in a spherical micelle with a maximum diameter size of 25 nm. Improved stability under acidic conditions and a controlled drug release was observed for the prepared micelle by PEG10 compared to the PEG2 unit [91]. Nelfinavir mesylate (NFM), an antiretroviral drug, is a HIV-1 protease inhibitor which also has reported antiviral activities toward SARS-CoV and SARS-CoV-2 [92]. A short half-life of 3.5 to 5 h and its lipophilic nature are two major disadvantages for oral administration consideration. Loading NFM by mixed micelles (with an average diameter size of 104.1 nm) composed of pluronic F127 and D-α-tocopheryl polyethylene glycol 1000 succinate (TPGS) improved drug loading, entrapment efficiency, bioavailability, sustained drug release and biodegradability [93].

**Figure 5:**
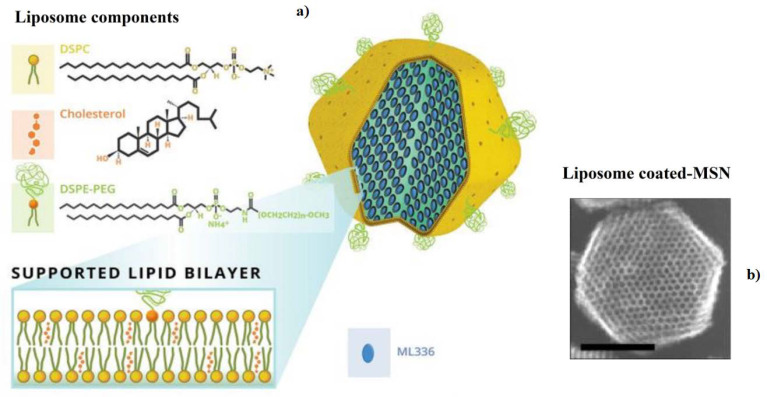
(**a**) Schematic and (**b**) TEM images of liposome-coated mesoporous silica nanoparticles (MSNs) with loading ML336 antiviral drug (scale bar = 50 nm) reproduced with permission from [103].

The use of essential oils or various extracts of plants to treat microbial infections has a long history in traditional and modern medicine. These properties have resulted from the existence of active metabolites in several parts of medicinal plants. For example, artemisinin and its derivative, dihydroartemisinin, are two sesquiterpene lactone metabolites extracted from the Artemisia annua plant species with anti-parasitic effects [94,95]. Moreover, there are several reports of anti-infection properties in the case of other species of the Artemisia plant genus [96]. Potential antiviral activities of essential oils extracted from Artemisia arborescens toward herpes simplex virus type 1 (HSV-1) was evaluated by a liposomal formulation [97]. In the general sense, one strategy towards controlling and managing the pandemic is to design and formulate efficient vaccines against the virus. In the case of mRNA vaccines, a carrier system is strictly required in order to protect the sensitive structure of mRNA molecules and deliver the active agent to certain cells of the immune system (drug targeting) to induce the expected immunization. Amongst the available strategies for the encapsulation and targeting drugs and active agents are liposomes, nanoliposomes, solid lipid nanoparticles and tocosomes [98]. Using an antiviral sample, compound and derived lipids, as well as immuno-modulator lipids, are other strategies to synthesize novel antiviral carriers or vaccine-based liposomes [79]. As a viral mimic, a double-stranded RNA of polyinosinic–polycytidylic acid and poly-L-lysine (poly-ICLC) is utilized as a prophylactic and antiviral agent for viral infections [99]. Upregulation of the expression of β-, γ- and α-interferon has resulted from the poly-ICLC effect. Further antiviral activities against Dengue virus (DENV) were shown for the liposomal formulation of poly-ICLC compared to poly-ICLC alone by the higher expression of IFN-γ and the promotion of innate immunity [100]. As previously mentioned, loading antibody fragments on the surface of liposomes may be another way to functionalize actively targeted carriers. Coupling llama heavy-chain antibody fragments (Vhh) onto the PEGylated liposome surface via covalent and non-covalent bonds having dapivirine, a non-nucleoside reverse transcriptase inhibitor, resulted in a reduction of HIV replication in vitro. A strong binding affinity to HIV-1 envelope glycoprotein gp120 and reduced neutralization potency were observed for covalently linked Vhh compared with a non-covalent one. In this regard, free antibodies with non-covalent bonds demonstrated a higher number of Vhh to bind to gp120 [101]. The thin-film hydration technique was utilized to prepare EFV-loaded liposomes with average diameter sizes and significant encapsulation efficiencies of 411.1 nm and 98.86%, respectively. The high encapsulation efficiency of this formulation was obtained using the soybean lecithin in the bilayer of the liposome, which had a solubilizing effect on EFV [102]. Lipid compounds may be employed to coat other organic or inorganic NPs such as polymeric NPs, metal NPs (MNPs), metal oxide NPs (MONPs) and mesoporous silica NPs (MSNs). For instance, ML336, a benzamidine antiviral medicine, was encapsulated via liposome-coated MSNs against the Venezuelan equine encephalitis virus (VEEV) (Figure 5). In vitro inhibition of VEEV resulted from improved circulation time and biocompatibility, as well as with a sustained drug release (6.6 ± 1.3 μg/mg) over 24 h [103].

Adefovir dipivoxil, a nucleotide analogue to decrease DNA level of hepatitis B virus (HBV) in serum, was loaded on SLNs towards the HepG2.2.15 cell line derived from the chronic HBV infection. Prepared drug–SLN formulations with a mean size of 389.4 ± 166.5 nm displayed sustained drug release. These formulations also displayed values of 15.32 ± 2.58% and 3.06 ± 0.51% for drug entrapment efficiency and drug loading, respectively [104]. The LDC form (PEGylated lipid–indinavir NPs) was activated by binding peptides of CD4-BP2 and CD4-BP4 to target CD4+ cells. Efficient drug delivery and indinavir release at acidic conditions (pH = 5) of endosome were explained as a pH-dependent drug release model for these carriers [105].

## 6. Application of Lectins in Micelle and Liposome Formulation

In this section, the functionalization of micelles and liposomes by several types of lectin is presented for non-viral infections because of the lack of this modification for antiviral purposes. Therefore, these examples can be useful to prepare novel formulations of vaccines or antiviral agents by lectin-modified micelles and liposomes in future investigations. To date, there is a lack of studies regarding modification of micelles by lectin. As one promising example, concanavalin A (Con A) lectin was conjugated to poly(ε-caprolactone)-block-glycopolymer micelles to improve mucoadhesiveness [106]. However, lectin modification of liposomes is more efficient owing to the stability of their bilayer structure compared to a micelle. As illustrated in Table 1, several interactions between liposomal components and lectins were used to prepare lectin-modified liposomes.

## 7. Conclusions

The commonly exploited micro- and nano-vesicles with a single bilayer leaflet for passive and active drug delivery applications are liposomes. In this regard, promising cell-binding drug carriers in the delivery of antiviral agents and vaccines can be obtained by actively using targeted micro- and nano-liposomes. The use of lipids with immuno-stimulatory effects is one approach to prepare efficient vaccines. The ability of lectins, particularly herbal lectins, to bind carbohydrate components of viruses may be a suitable strategy for targeting coronaviruses. To this end, MBL related to red alga has received considerable attention owing to its strong binding with the spike glycoprotein of coronaviruses. It is worth mentioning that there are not enough investigations about lectin-modified liposomal formulations for coronaviruses. Therefore, in future studies, it is expected that new generations of the cell-binding liposomes based on lectin functionalization with the capacity to encapsulate antiviral agents on the surface, bilayer, and aqueous sections will be investigated. It is also worth noting that other promising approaches, such as using hrsACE2 to functionalize the liposomal or micellar surface, can also improve this type of active carrier. Moreover, the cell type employed to grow the virus stocks is a critical factor to determine the inhibition capacity of micellar and liposomal formulations towards coronaviruses. For instance, in physiological conditions of the body, considering the function of the immune system is an important factor in understanding of antiviral activities of these formulations. Additionally, the testing of various types of lectins is vital because of the altering of the N-glycosylation pattern of coronavirus glycoproteins by genetic mutations. Therefore, many curious in vitro and in vivo studies are required to achieve these objectives.

## Figures and Tables

**Table 1:** Applications of several types of lectin in liposome formulations.

Sources of Lectin	Application in Liposome	References
Wheat germ agglutinin (WGA) lectin of *Triticum vulgare* plant	Incorporation of the lectin in the bilayer liposome for potential oral vaccine carriers.	[107]
WGA lectin of *T. vulgare* plant	WGA-modified liposome with the ability to bind the N-acetylglucosamine was used as an aerosol formulation for drug delivery to human alveolar epithelial cells.	[108]
Con A, WGA, and soybean agglutinin (SBA) extracted from *Canavalia ensifomis*, *T. vulgare*, and *Glycine max* plant species, respectively	Coupling lectins with liposomes with avidin/biotin technology. Con A, WGA, and SBA have carbohydrates specificity for α-D-mannose or α-D-glucose, N-acetyl-glucosamine oligomers and N-acetyl I-galactosamine, respectively.	[109]
*Cratylia mollis* plant species	Encapsulation of the lectin in the liposome.	[110]
WGA lectin of *T. vulgare* plant	Phosphatidylethanolamine in the bilayer was covalently bound to the lectin to prepare the WGA-modified liposome.	[111]
Lectin extracted from *Lotus tetragonolobus* plant species	Formation of oleic acid–lectin conjugation in the phospholipid bilayer of liposomes with the ability to bind glycans having alpha-1,2-linked fucose.	[112]
Tarin lectin of *Colocasia esculenta* plant species	The lectin was encapsulated in the aqueous phase of a liposome. Tarin demonstrated a promising binding site for complex and high-mannose N-glycan chains related to viral surface antigens. This lectin can help a host to recover from infections by the stimulation of innate and adaptive immune responses.	[113,114]
Lectin extracted from *Bauhinia variegate* plant species	Based on FTIR and NMR analyses, there was a strong interaction between the lectin and phosphatidylcholine of the liposome in the outer and inner polar surface of the liposome. The rotational motion of the lipid group was restricted by this interaction.	[115]
Lectin of *T. vulgare* plant, WGA	Interaction of the lectin with the bilayer of the liposome resulted in cytoadhesive and cytoinvasive effects, as well as increased permeability in the cell membrane and cellular uptake compared with the non-lectin liposomes for oral epithelial cells	[116]
WGA-N-glutaryl-phosphatidylethanolamine	Protection of the drug against enzyme degradation in vitro as well as higher stabilization of WGA-modified SLNs compared to bare SLNs.	[117]
